# 

**Published:** 1842-03

**Authors:** 


### WESTERN JOURNAL

OF

### MEDICINE AND SURGERY.

### EDITORS.

---

DANIEL DRAKE, M. D.,

PROFESSOR OF PATHOLOGICAL ANATOMY AND CLINICAL MEDICINE IN THE MEDICAL INSTITUTE OF LOUISVILLE:

LUNSFORD P. YANDELL, M. D.,

PROFESSOR OF CHEMISTRY AND PHARMACY IN THE SAME:

THOMAS W. COLESCOTT, M. D.

### RECEIPTS

### *For the Medical Journal for February*, 1842.

---

| | |
|---|---|
| Dr. A. H. Wall, Ruddle's Mills, Ky. | $10 00 |
| W. N. Gaither, Cerulean Springs, Ky. | 5 00 |
| J. Sweney, Dripping Spring, Ky. | 5 00 |
| M. D. Wilson, Dukedom, Tenn. | 5 00 |
| J. Harden, Horsewell, Ky. | 10 00 |
| Bradley, Waterford, Pa. | 3 00 |
| J. G. Jones, Covington, Ia. | 5 00 |
| C. E. Williams, Paris, Ky. | 5 00 |
| W. B. Holmes, Granville, Tenn,. | 5 00 |
| J. A Shuttleworth, Campbellsville, Ky. | 5 00 |
| M. L. Reid, Hagerstown, Ia. | 5 00 |
| J. W. Stout. Germantown, Tenn., | 5 00 |
| J. Howell, Hamilton, O. | 5 00 |
| J. H. Kilbey, Griggsville, Ill. | 5 00 |
| L. Loop, Thorntown, Ia., | 5 00 |
| J. M. Chamberlain, Harmansburgh, Pa. | 5 00 |
| J. T. Sharp, Cambridge City, Ia. | 5 00 |
| W. H. Jenkins, Dresden, Tenn. | 5 00 |
| Walworth, Vernon, O. | 5 00 |
| W. J. Berry, Hartford, Ky,, | 5 00 |
| J Thistle, Natchez, Miss, | 5 00 |
| Drs, A, H, & J, Pollock, Germantown, Ky, | 5 00 |
| Dr, T. F. Titus, De Kalb, Texas, | 5 00 |
| T D, Isom, Oxfold, Miss | 5 00 |
| G. R, Grant, Jacksonville, Ala, | 5 00 |
| A, T, Boyd, Bigbyville, Tenn, | 5 00 |
| D. G. Gregory, Elm Grove, Mo. | 5 00 |
| R. J. Swearingen, Pickensville, Ala,, | 5 00 |
| J, Mitchell, Washington, Miss, | 5 00 |
| E. McAllister, do do | 5 00 |
| A Moore, Woodville, Ala, | 5 00 |

